# Echoes of Emotions Past: How Neuromodulators Determine What We Recollect

**DOI:** 10.1523/ENEURO.0108-18.2019

**Published:** 2019-03-15

**Authors:** David Clewett, Vishnu P. Murty

**Affiliations:** 1 Department of Psychology, New York University, New York City, New York, 10003; 2 Department of Psychology, Temple University, Philadelphia, PA, 19122

**Keywords:** context, dopamine, emotion, episodic memory, norepinephrine, recollection

## Abstract

We tend to re-live emotional experiences more richly in memory than more mundane experiences. According to one recent neurocognitive model of emotional memory, negative events may be encoded with a larger amount of sensory information than neutral and positive events. As a result, there may be more perceptual information available to reconstruct these events at retrieval, leading to memory reinstatement patterns that correspond with greater memory vividness and sense of recollection for negative events. In this commentary, we offer an alternative perspective on how emotion may influence such sensory cortex reinstatement that focuses on engagement of the noradrenergic (NE) and dopaminergic (DA) systems rather than valence. Specifically, we propose that arousal-related locus coeruleus-norepinephrine (LC-NE) system activation promotes the prioritization of the most salient features of an emotional experience in memory. Thus, a select few details may drive lower-level sensory cortical activity and a stronger sense of recollection for arousing events. By contrast, states of high behavioral activation, including novelty-seeking and exploration, may recruit the DA system to broaden the scope of cognitive processing and integrate multiple event aspects in memory. These more integrated memory representations may be reflected in higher-order cortical reinstatement at retrieval. Thus, the balance between activation in these neuromodulatory systems at encoding, rather than the valence of the event, may ultimately determine the quality of emotional memory recollection and neural reinstatement.

## Significance Statement

We tend to re-live emotional events more richly in memory than mundane experiences. Recent research suggests that emotional memory enhancements, particularly for negative events, emerge from greater reactivation of sensory information during retrieval. Here, we offer an account of how emotion influences such sensory cortex reinstatement that focuses on how activation of the noradrenergic (NE) and dopaminergic (DA) systems may impact the quality of recollection. We propose that NE system activation promotes the prioritization of the most salient features of an event in memory, whereas DA system activation promotes the integration of multiple event features in memory. Thus, the balance between activation in these systems might determine how we encode and later re-experience our most significant events.

## 

Emotional events are resistant to forgetting and are vividly remembered (LaBar and Cabeza, 2006; Markovic et al., 2014; Yonelinas and Ritchey, 2015). This privileged status of emotional experiences in memory is highly adaptive, because it promotes future behaviors that help us avoid threat and maximize well-being (Shohamy and Adcock, 2010; Eaton and Anderson, 2018). Years of psychology and neuroscience research have uncovered adaptive brain mechanisms that preferentially support the formation and storage of emotional memories. However, these neurocognitive models have focused disproportionately on earlier stages of memory processing, including encoding and consolidation (for review, see McGaugh, 2004; LaBar and Cabeza, 2006), with less attention being given to emotion’s downstream effects on later memory retrieval.

In their recent Negative Emotional Valence Enhances Recapitulation (NEVER) model, Bowen et al. (2018) theorize a significant role for retrieval-related processes in facilitating emotional memory (Bowen et al., 2018). Specifically, they propose that negative memories contain more sensory information from the original event than both neutral and positive memories. These perceptually-rich mental representations are then reinstated in the brain during retrieval, resulting in more detailed and vivid memories for negative events. The authors also suggest that negative valence strengthens the links between different stages of episodic memory, such that more sensory-focused encoding leads to preferential reactivation of these perceptual details during offline consolidation and, consequently, later recollection. The NEVER model thereby differentiates itself from prior models of emotional memory by: (1) highlighting a prominent role for retrieval-related sensory processing in facilitating emotional memory, and (2) emphasizing that these mnemonic advantages are specific to negative events.

Although this framework offers a compelling account of the phenomenology of emotional memory recollection, it is somewhat incomplete. Here, we propose some important considerations for the NEVER model and discuss some methodological caveats that warrant further attention. Building on this critique, we offer another perspective of how emotion influences retrieval-related sensory cortex reinstatement, or “recapitulation,” that focuses on how activity in the noradrenergic (NE) and, possibly, dopaminergic (DA) systems may impact emotional memory formation and the quality of recollection. Our neuromodulation-based model expands upon NEVER by clarifying which aspects of emotional experiences are being recapitulated during retrieval. It also specifies multiple neural mechanisms by which neural reinstatement may occur, including several factors, such as physiological arousal and behavioral activation, that determine these differential patterns of engagement. By filling in these knowledge gaps, we aim to provide a more holistic view of how emotion influences what we remember about our most significant experiences.

### Brain mechanisms that support the recollection of past events

When memories are brought to mind, it can often feel as if those events are being re-experienced in the moment (Tulving and Markowitsch, 1998). Seminal neurocognitive models of recollection propose that the extent to which we re-live prior events may be driven by greater correspondence between retrieval-related brain activity and the neural processes that were engaged at encoding (McClelland et al., 1995; Danker and Anderson, 2010). At the neural level, this idea is supported by neuroimaging research showing that successfully retrieving prior episodes is associated with reactivation of neural memory and sensory traces first laid down at encoding (Nyberg et al., 2000; Wheeler et al., 2000; Danker and Anderson, 2010; Mack and Preston, 2016; Tompary et al., 2016; Danker et al., 2017).

Emotional memories tend to be highly vivid, suggesting that they may contain richer sensory information that is available for reconstruction, or recollection, during later retrieval. Behavioral evidence suggests that negative emotional events, in particular, are remembered with greater perceptual detail than neutral and sometimes positive events (Kensinger, 2007). Supporting this notion, human fMRI studies show that encoding negative experiences relates to greater activity in perceptual processing regions, including visual cortex, suggesting that negative memories may contain more perceptual details than other memories (Kensinger et al., 2007a,b; Mickley and Kensinger, 2008).

Until recently, the majority of research has focused on how emotional memories garner greater attention than neutral experiences and are preferentially processed during encoding. This has left an important knowledge gap concerning how emotion impacts memory retrieval processes downstream, which has important implications for the wellbeing of both healthy individuals and individuals with emotion or anxiety disorders when they re-live their most aversive event. In this section, we outline key predictions of the NEVER model, which was proposed to fill this critical gap.

### The NEVER model: core tenets and primary empirical evidence

According to the NEVER model, emotion-related biases in perceptual processing may re-emerge during retrieval, leading to a more vivid sense of recollection. Building on earlier investigations targeting neural recapitulation effects with neutral stimuli, Bowen et al. (2018) hypothesized that there is greater encoding-retrieval overlap in brain activity within early sensory processing regions, particularly for negative stimuli.

In an initial test of this idea, Kark and Kensinger (2015) used fMRI to examine brain activity patterns associated with encoding and retrieving emotional and neutral experiences. During encoding, participants viewed colored photographs of negative, positive, and neutral scenes along with corresponding black-and-white line drawing of those pictures. During retrieval, participants had to decide whether line drawings were previously associated with a colored image or whether they had never been seen before. One advantage of this retrieval manipulation is that it avoids reintroducing strong arousal/emotional states by using the emotional associate as the memory cue. Thus, any patterns of reinstatement can be more clearly interpreted as mental reactivation as opposed to purely sensory properties of the cue.

**Figure 1.**
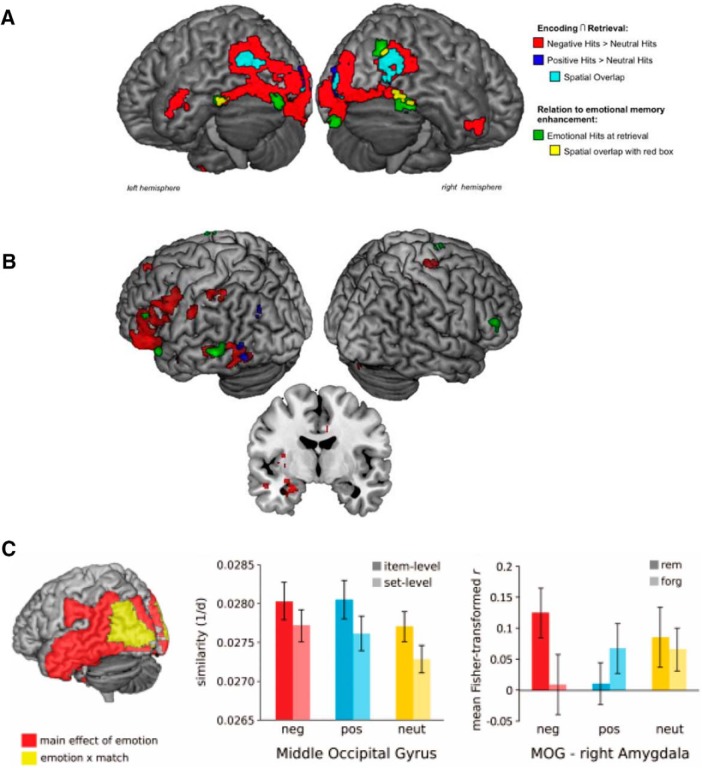
Empirical evidence of emotion-enhanced sensory cortex and amygdala recapitulation during memory retrieval. ***A***, Conjunction fMRI analyses of brain activity associated with successful memory during encoding and retrieval revealed significant spatial overlap (i.e., recapitulation) in ventral temporo-occipital cortex for negative and positive emotional images (red, blue and turquoise clusters). The spatial extent of recapitulation across the visual stream was greater for negative (red clusters) than positive emotional stimuli (blue clusters). Areas associated with emotion-related recapitulation were also correlated with emotional enhancements (not differentiated by valence) in behavior (data and figure from Kark and Kensinger, 2015). ***B***, Extending these findings, one recent fMRI study using encoding-retrieval conjunction analyses revealed greater extent of recollection-specific activity (i.e., “remember hits” vs “know hits”) across PFC, temporal cortex, and occipital cortex for negative images (red clusters). Similar neural recapitulation patterns were observed for positive (blue clusters) and neutral stimuli (green clusters) in ventral occipito-temporal cortex, but to a quantitatively lesser extent than negative stimuli. Recollection of negative valence stimuli was also associated with enhanced recapitulation activity in the amygdala. Because neutral word cues were used to cue “remember” or “know” memory decisions, these neural patterns are interpreted as evidence of the recapitulation of the perceptual content of emotional memories (data and figure from Bowen and Kensinger, 2017). ***C***, In a separate fMRI study, Ritchey et al. (2013) used pattern similarity analyses to examine correspondence between item- versus set-level activation patterns during encoding and retrieval of positive, negative, and neutral stimuli. The results revealed similar patterns of memory-related recapitulation in middle occipital gyrus for positive (blue bars) and negative (red bars) versus neutral stimuli (yellow bars) for item-specific representations. Further, the degree of encoding-retrieval similarity in middle occipital gyrus was positively correlated with retrieval-related activity in the amygdala, but only for negative valence stimuli (data and figure from Ritchey et al., 2013).

**Figure 2.**
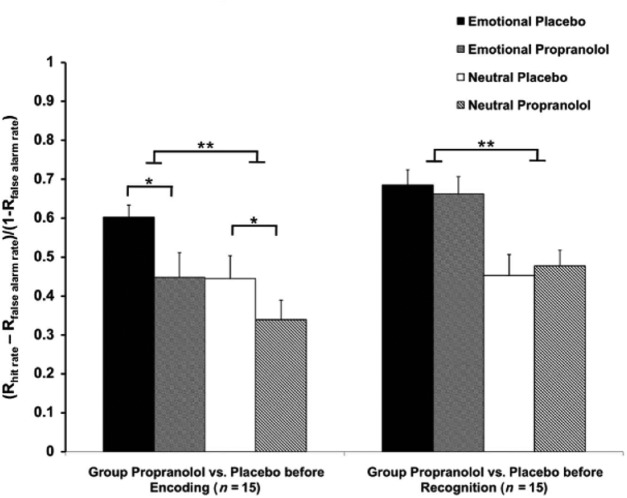
β-Adrenoreceptor blockade impairs emotional memory recollection at encoding but not at retrieval. Emotion led to enhanced recollection of photographs when memory was probed 24 h after learning. Administration of 80 mg of propranolol led to decreased recollection for both emotional and neutral stimuli, whereas propranolol had no effect on emotional or neutral recollection (data and figure from Rimmele et al., 2016). Asterisks indicate statistical significance.

Although there were no emotional memory enhancements in recognition memory, conjunction analyses performed on the fMRI data revealed overlapping patterns of activation between encoding and retrieval. These analyses revealed three key findings: (1) in large portions of lateral occipito-temporal cortex, there was large spatial overlap across encoding and retrieval for successful memory-related brain activity for both positive and negative images (Fig. 1*A*); (2) in inferior temporal gyrus, there was significantly greater spatial overlap between the encoding and retrieval of negative versus positive images; and (3) for negative items, there was greater spatial overlap between encoding and retrieval in fusiform gyrus, a region that was also more functionally coupled with amygdala activity during successful encoding. These findings suggest that negative emotional memories may be recapitulated to a larger extent than positive memories in regions within ventral occipito-temporal cortex, and this sensory recapitulation of negative stimuli may be mediated by amygdala modulation at encoding.

In a more recent study, Bowen and Kensinger (2017) designed an fMRI paradigm that could more directly test this relationship between emotional memory recollection and neural recapitulation. During encoding, participants viewed neutral words that were paired with negative, positive, and neutral images. These images consisted of either faces or scenes. During retrieval, participants were shown the neutral word cue and were asked to retrieve the accompanying picture. Like the prior study, the strength of this approach is that reactivation differences between valences cannot be attributed to sensory activation driven by the emotional images themselves. Rather, the word cues are equally devoid of strong visual input. Behaviorally, memory was enhanced for neutral words paired with negative scenes compared to positive or neutral scenes; however, there was no valence differences for words paired with emotional or neutral faces. Conjunction fMRI analyses revealed qualitatively greater recollection-related encoding-retrieval spatial overlap in visual occipito-temporal cortical activity and amygdala for negative contexts than positive and neutral contexts (Fig. 1*B*), although this pattern was not seen in category-selective visual cortex. This pattern of findings remained when the authors controlled for stimulus arousal across valence (comparing positive scenes vs negative faces) and lower-level stimulus features (comparing positive vs negative faces).

Together, the findings from these fMRI studies lend initial support to the idea that when negative emotional memories are successfully remembered (as, on average, there were no behavioral effects of emotion on memory), they may be recollected with greater perceptual information than positive and neutral memories. Inspired by these findings, the NEVER model was put forth to address the underappreciated role of emotional valence on episodic memory. The primary aims of this brain-based model are to: (1) shift the focus of emotional memory mechanisms beyond regions traditionally implicated in emotional memory, including the amygdala and hippocampus (Dolcos et al., 2004; Ritchey et al., 2008; Murty et al., 2010), to capture the local reinstatement of sensory-rich negative memory representations at retrieval; (2) encourage a broader appreciation of non-arousal-related factors that may enhance memory vividness and recollection; and (3) place a larger emphasis on how different stages of episodic memory processing interact, with a particular focus on how encoding biases relate to subsequent patterns of post-encoding memory reactivation and retrieval.

### A new perspective of emotional memory and neural reinstatement

The NEVER model offers an interesting account of the neural factors that facilitate emotional memory and its persistence. But arguably, the bulk of the literature still implicates physiological arousal as a driving force behind the modulation of recollection effects. Indeed, the critical role of physiological arousal in modulating episodic memory remains a central feature of many neurocognitive theories of emotional memory (see Table 1 in Bowen et al., 2018). While it is possible that valence effects are still at play, there are notable caveats in NEVER’s empirical studies that need to be addressed, including de-confounding the effects of valence and arousal on neural recapitulation. Importantly, the effects of positive valence on recollection are quite variable (see Table 2 in Bowen et al., 2018). For instance, in some cases, behavioral work shows that positive events are also encoded with great perceptual detail, perhaps even more than negative stimuli (Talarico et al., 2009).

A challenge to understanding the role of valence in emotional memory, has been operationalizing the influence of arousal on the formation of positive memories. Despite efforts to experimentally control for arousal differences between positive and negative emotional stimuli, participants’ ratings sometimes deviate from normative arousal ratings [e.g., international affective picture system (IAPS) images; Lang and Bradley, 2007]. Due to this, it may be the case that arousal-related confounds cloud the interpretation of valence differences that have been reported across many studies of emotional memory. In prior research, these arousal-related confounds have emerged in several ways. First, in some cases, arousal was not experimentally controlled between positive and negative emotional stimuli (Maratos et al., 2001). In other cases, differences in subjects’ self-reported arousal persisted even though arousal levels were matched between positive and negative emotional stimuli beforehand (Kark and Kensinger, 2015). Because negative stimuli tend to be much more arousing than positive stimuli, one possibility is that valence differences emerge due to arousal levels being relatively moderate. In other words, to be able to equate arousal levels across valences, the arousal level of selected negative stimuli may be relatively low and therefore fail to capture a robust effect of high-arousal states on declarative memory. Finally, in one fMRI study, stimuli drawn from different visual categories (i.e., negative faces, and positive scenes) had to be compared to control for arousal (Bowen and Kensinger, 2017).

Based on issues like these, we believe it is premature to rule out the possibility that arousal mechanisms, rather than valence, mediate emotion-related effects on reactivation in early sensory cortex. This idea is further reinforced by a prior fMRI study reporting equivalent levels of sensory recapitulation during retrieval for both positive and negative memoranda in general. But valence differences emerged when examining amygdala modulation, which may reflect variability in arousal across emotional valences (Ritchey et al., 2013; Fig. 1*C*).

To address these issues, we propose a new model that considers emotional memory reinstatement processes within the broader context of the emotion, episodic memory, and motivation literatures. Our goal is to move beyond a valence-based interpretation of emotional memory recollection, per se, to encompass other cognitive and emotional factors that impact various stages of episodic memory. The main points of our framework can be summarized as follows:

(1) Sympathetic arousal, rather than negative valence, drives selective memory of prioritized and item-intrinsic features during encoding, leading to enhanced subjective sense of recollection and memory vividness. This cognitive narrowing effect also results in greater reinstatement patterns in relatively lower-level sensory cortical regions during retrieval. Further, this process may be driven by arousal-related NE system activity and amygdala activity at encoding and consolidation, as well as by amygdala (re)activation at retrieval.

(2) Behavioral activation, defined as increased behavioral vigor or novelty-seeking, may engage alternative mechanisms that reflect the formation of integrative, flexible memory representations. This memory process may be signified by reinstatement effects in relatively higher-order cortex, such as anterior portions of the ventral visual stream as well as lateral and medial prefrontal cortex (PFC). One possibility is that mesolimbic DA system activation supports these more integrative forms of memory and high-order reactivation.

(3) Focusing on arousal and behavioral activation rather than valence may provide a better schema for understanding enhanced neural reinstatement patterns for emotional memories. Critically, emotional valence may be inter-related with arousal and behavioral activation, such that negative stimuli are biased towards eliciting greater arousal and positive stimuli are biased towards eliciting behavioral activation. However, in many behavioral contexts, negative stimuli might evoke behavioral activation, and positive stimuli may elicit high degrees of arousal. Thus, our goal is to move beyond valence effects to consider alternative factors that shape patterns of neural reactivation/reinstatement and its manifestation of memory.

### What types of sensory information are reinstated during emotional memory retrieval?

The NEVER model proposes that sensory features of a negative emotional event are reactivated in sensory cortex during retrieval, with the implication that this recapitulation brings to mind the details and vividness of the original event. We broaden this interpretation by proposing that these recapitulation effects reflect certain aspects of the original event in which only the most salient item and episodic details are stored in memory and later reinstated. Current data suggest that this process may be predominantly driven by increased emotional arousal.

A large body of research shows that recollection, defined as remembering an item with accompanying episodic information, is better for emotional experiences than for neutral experiences. However, it has long been known that the impact of emotion on cognition is highly selective and does not benefit all aspects of episodic memory (Anderson et al., 2006; Rimmele et al., 2011; Bisby and Burgess, 2013; Steinmetz and Kensinger, 2013; Madan et al., 2017). This issue raises the critical question of *which* episodic details of emotional experiences are being reinstated and recollected at retrieval.

Studies that query source memory (e.g., an item’s timing, location, or perceptual features) may provide some clues as to the episodic content of emotional memories. A wealth of evidence suggests that memory is selectively enhanced for emotional items and their intrinsic details (Yonelinas and Ritchey, 2015), such as their location or color, whereas memory for spatially or temporally adjacent contextual information is impaired (Christianson and Loftus, 1991; Kensinger, 2007; Mather, 2007; Touryan et al., 2007; Mather and Sutherland, 2011; Steinmetz and Kensinger, 2013; Bisby and Burgess, 2017). Further, prior work has linked such emotional memory selectivity to increased arousal levels rather than valence (Mather and Nesmith, 2008), consistent with the idea that arousal focuses limited mental resources towards processing a select few details of an emotional event (Easterbrook, 1959).

One possibility is the strength of these specific memory traces modulates subjective aspects of emotional memories. Emotion enhances the subjective sense of remembering, such that participants tend to report higher subjective confidence and vividness for high arousal, emotional memories (Ochsner, 2000; Kensinger and Corkin, 2003; Sharot et al., 2004; Touryan et al., 2007; Ritchey et al., 2008; Todd et al., 2013b). Yet this increased confidence does not always correspond with greater memory accuracy, at least for all aspects of an emotional experience. Rather, subjective memory confidence appears to relate specifically to successful retrieval of emotional item-intrinsic details, such as the location and timing of an emotional stimulus (Rimmele et al., 2012) and not more peripheral sensory information, such as the surrounding context (Rimmele et al., 2011).

Considering that highly confident memory decisions are closely tied to memory for emotional item-intrinsic information, it may be the case that enhanced recollection for emotional events is not driven by the amount of sensory details that are recovered. Instead, vividly re-experiencing a prior emotional event could be driven by especially strong memory representations of select details (for a similar proposal, see Kensinger, 2009). Indeed, higher subjective memory response encountered after an arousing negative event have been linked to enhanced memory for intrinsic sensory features (e.g., color) of those neutral items (Xie and Zhang, 2017). Eye-tracking evidence also shows that recollection judgements for high-arousal negative images are predicted by more clustered rather than distributed fixations during encoding, suggesting that specific features of these events drive subsequent recollection (Sharot et al., 2008). Together, these data are consistent with evidence that emotional arousal amplifies the effects of priority (e.g., goal-relevance, bottom-up salience) in perception and memory, leading to the selective encoding of highly salient information (Mather and Sutherland, 2011; Sakaki et al., 2014).

In sum, we argue that for high arousal memoranda, which are often tested in the domain of negative valence, the subjective sense of remembering is driven by a narrowing of attention towards only the most central, emotionally-laden details of an experience. We note that this more focused cognitive processing induced by arousal should incorporate neutral information insofar as those neutral associates are task-relevant and actively prioritized under emotional circumstances. In these situations, neutral cues will be bound more strongly to their associated emotional items in memory, and can act as an effective trigger to recall (and/or recapitulate) emotional events (for review, see Mather and Sutherland, 2011; Murray and Kensinger, 2013; Sakaki et al., 2014). For instance, both positive and negative background scenes can enhance memory for foreground neutral objects when participants are instructed to unitize those inputs (e.g., imagine them interacting) during encoding (Ventura-Bort et al., 2016).

To interpret the true nature of emotion-related sensory recapitulation, then, it is critical to consider where attention is allocated during encoding, and, second, whether participants’ memory is assessed for the salient or non-salient features of that emotional event. This feature of emotional memory selectivity suggests that sensory recapitulation reflects a biased representation of the most salient event features, perhaps at the expense of the surrounding or irrelevant perceptual information.

### Is the preferential processing and retrieval of emotional item-intrinsic information dependent on the amygdala?

Next, we consider how emotional arousal may contribute to greater episodic memory selectivity during emotional memory. Much research implicates amygdala processes in modulating the quality of emotional memory recollection, particularly by promoting the encoding of specific details of arousing events (Sharot et al., 2004; Phelps and Sharot, 2008). Greater amygdala activity at encoding has been specifically linked to successful item recognition and higher subjective memory vividness for positive and negative images, but not to the successful memory of peripheral contextual information (Dougal et al., 2007; Kensinger et al., 2007a,b). Lesion data corroborate this role of the amygdala in selective memory processes. Patients with amygdala damage show selective memory deficits for the central but not peripheral details of an emotional event (Adolphs et al., 2001, 2005; Yonelinas and Ritchey, 2015). Thus, amygdala activity at encoding may lead to more vivid recollections by forming especially strong memories of highly specific aspects of an emotional event.

At the network level, this memory selectivity may result from the amygdala modulating early sensory cortical activity (Todd et al., 2012, 2013b). For instance, for high-arousal negative stimuli, greater encoding-related amygdala functional connectivity with the fusiform gyrus, a region associated with processing item-exemplar information (Garoff-Eaton et al., 2006), was also associated with greater sensory recapitulation (encoding-retrieval overlap) in fusiform gyrus (Kark and Kensinger, 2015). This suggests a link between early amygdala tuning processes in sensory cortex during emotional memory formation and local sensory cortex reinstatement effects.

The notion that negative valence may modulate these amygdala effects is partially motivated by fMRI evidence using effective (e.g., directional) connectivity analyses (Mickley Steinmetz et al., 2010). This study showed that patterns of amygdala modulation at encoding diverge at different levels of arousal in a valence-dependent manner. Specifically, amygdala modulation of middle occipital gyrus activity was increased for high-arousal negative stimuli but decreased for high-arousal positive stimuli. But it is important to note that the opposite valence-dependent flip was observed in the amygdala’s modulation of fusiform gyrus activity: whereas the amygdala’s influence over activity in this higher-order sensory region was increased for high-arousal positive images, it was decreased for high-arousal negative images. This again raises the question of what types of sensory information are being recapitulated by amygdala engagement. Without querying which specific episodic details are being remembered and linking this to brain activation patterns, it is difficult to interpret what non-category-selective patterns of cortical activation mean for models of sensory recapitulation.

Emotion-related encoding biases may also propagate downstream and manifest in patterns of post-encoding functional connectivity, particularly when arousal levels remain elevated. The way emotional information is encoded therefore determines how emotional experiences are subsequently consolidated and retrieved. The amygdala also appears to shape emotional memory in this regard, either exhibiting increased local activity or more distributed patterns of modulation during the encoding, consolidation, and retrieval of emotional memories (Buchanan, 2007). Fear learning paradigms, for example, offer a robust way of manipulating arousal responses that facilitate memory. Across several fMRI studies, increases in amygdala functional connectivity with the hippocampus (de Voogd et al., 2016; Hermans et al., 2017) and category-selective cortex (Hermans et al., 2017) have been shown to persist into post-conditioning periods of wakeful rest. These connectivity patterns also predicted spontaneous fear recovery 24 h later. The findings lend support to influential theories positing that the amygdala modulates systems-level consolidation processes that enhances the long-term retention of emotionally arousing memories (McGaugh and Roozendaal, 2002; McGaugh, 2000).

Research also suggests that amygdala activity contributes to emotional memory recollection, in part through reinstatement (Bowen and Kensinger, 2017; Fig. 1*B*). One possibility, however, is that this recapitulation relates more closely to high arousal rather than negative valence. For instance, amygdala activity during memory retrieval tracks individual self-reports of arousal on a stimulus-by-stimulus basis (Dew et al., 2014). This suggests arousal-related activation of the amygdala promotes more sensory-focused attention and encoding processes in a valence-invariant fashion. The specific conditions that give rise to valence differences at retrieval require additional research, given that amygdala activity at retrieval has also been reported for positive stimuli (Buchanan, 2007).

Amygdala processes might also modulate the subjective quality of emotional memory recollection during the retrieval act itself, but not necessarily memory accuracy (Sharot et al., 2004). In fact, amygdala activity may give rise to a false sense of remembering, especially when participants must retrieve or recollect contextual information that is peripheral to the to-be-encoded item, such as a neutral or arousing negative background (Takashima et al., 2016). Across multiple fMRI studies, amygdala and sensory cortex activity in response to contexts or items deemed arousing, including negative emotional items, are either unrelated to or negatively associated with subsequent source memory (Smith et al., 2006; Takashima et al., 2016). These findings are consistent with behavioral work showing that source memory is impaired for information that is peripheral to an emotional stimulus, despite individuals endorsing an emotional stimulus as being remembered (Rimmele et al., 2011). Thus, some aspects of retrieval-related sensory cortex and amygdala activity may represent noisy or inaccurate cognitive/sensory representations, especially for peripheral details that may have been poorly encoded to begin with.

In summary, current findings point to a role of arousal-driven amygdala activation and modulation in encoding, consolidating, and retrieving emotionally arousing memories. However, neural reinstatement effects may be more nuanced in that they support the accurate recollection of prioritized sensory features but not less salient peripheral features. These results also underscore the importance of querying recollection for both central and peripheral details of an emotional event, and linking different aspect of memory performance to brain activity patterns across different stages of episodic memory.

### Does neuromodulation explain differences in the prioritization of sensory features in emotional memory?

To better understand how emotion influences neural reinstatement processes in the brain, we propose that it is important to consider the valence-invariant role of neuromodulatory arousal systems in supporting emotional memory encoding. It is well established that arousal-induced activation of the locus coeruleus-norepinephrine (LC-NE) system contributes to the superior processing, encoding, and consolidation of emotional experiences in long-term episodic memory (Roozendaal et al., 1996, 2008; Southwick et al., 2002; McGaugh, 2004; Strange and Dolan, 2004; Segal and Cahill, 2009). Through its diffuse neuronal projections to most of the brain, the LC is well positioned to modulate sensory cortical and amygdala processes during highly arousing and salient events (Berridge and Waterhouse, 2003; Sara, 2009). Such modulation under arousal may be particularly important in shaping the contents of memory during encoding. Human memory studies utilizing pharmacological and genetic approaches have shown that putative increases in NE tone at encoding but not retrieval enhance the subjective recollection and vividness of emotional memory later on (Rimmele et al., 2016; Fig. 2).

Research suggests that amygdala-centric mechanism of emotional memory may be in large part driven by the release of NE both during emotional encoding and consolidation (McGaugh, 2004, 2015; Strange and Dolan, 2004). But these selective arousal-related memory enhancements may come at the expense of processing spatially or temporally adjacent neutral information, suggesting that NE modulation of the amygdala biases attention and memory processes to favor emotional over neutral information (Strange and Dolan, 2004; Markovic et al., 2014). These empirical findings lend strong support to influential neurobiological models of emotional memory, which posit that NE signaling under arousal biases the amygdala to preferentially enhance encoding and long-term storage of emotionally salient mental representations in the sensory cortex (e.g., visual cortex), the medial temporal lobe (e.g., entorhinal cortex), and hippocampus (McGaugh, 2004; Roozendaal et al., 2008; Tully and Bolshakov, 2010; McIntyre et al., 2012; Markovic et al., 2014; Mather et al., 2016). Under emotional arousal, elevated levels of NE also enhance the vividness of perception, in part through increased amygdala influence over sensory cortex activity (Todd et al., 2013b, 2015).

Activation of the LC-NE system has also been observed during emotional memory retrieval. In one fMRI study, greater functional connectivity between the amygdala and LC was associated with successful retrieval of neutral items encoded in aversive contexts (Sterpenich et al., 2006). Further, activity in the LC activity during successful memory retrieval varied according to the level of item-specific arousal at encoding. This result is consistent with the idea that arousal-related modulation during initial processing has downstream consequences on emotional memory retrieval. Supporting this idea, pharmacologically blocking β-adrenoreceptors only impairs emotional memory recollection when administered prior to encoding but not prior to retrieval (Rimmele et al., 2016). Intriguingly, enhancing NE activity in the rodent amygdala following episodic-like memory training has been shown to preserve hippocampal-dependent retrieval and accurate memory 28 d later, suggesting that emotional memories may retain their vividness via NE in the amygdala (Atucha et al., 2017). Thus, without sustaining NE levels after emotional learning and during consolidation, arousal may not exert as strong of an effect on emotional memory.

Together, these findings suggest that the predominant mechanism driving arousal’s influence on emotional memory may reflect NE’s more general role in cognitive selectivity and attention. Recent theoretical and empirical work suggest that NE release biases both attention and memory to favor high priority (e.g., goal-relevant or perceptually salient) mental representations at the expense of lower priority information irrespective of valence (Lee et al., 2014, 2018; Markovic et al., 2014; Nielsen et al., 2015; Mather et al., 2016; Clewett et al., 2017a,b, 2018a). From this perspective, increased LC-NE activity under arousal promotes the selective encoding of contextual details that are within the focus of attention, binding only the most important aspects of an experience into long-term episodic memory.

These neurocognitive models also suggest that the vivid recollection of emotional events is not determined by negative valence, per se, but rather by synergistic interactions between arousal and any form of stimulus salience at the time NE is released (Mather et al., 2016). Combined rodent and human findings suggest that these strengthened processing-stage interactions may relate more to closely to arousal and LC-NE system activation rather than valence alone. Similar to negative stimuli, activity in the NE-LC system may support sensory recapitulation for positive memoranda. LC responses are not limited to exposure to negative events but also generalize to positive events that are highly arousing or motivating (Berridge and Waterhouse, 2003; Bouret and Sara, 2004; Sara, 2009; Bouret and Richmond, 2015; Todd et al., 2015). Supporting an arousal-based versus valence-based account of emotional memory encoding and retrieval, positive stimuli tend to show neurobehavioral effects similar to negative stimuli in states of putatively greater LC-NE engagement. For example, positive and negative events engage the amygdala to a similar degree under stress, an elevated state of arousal (Cousijn et al., 2010). Broadly, these results align with evidence that amygdala activity also supports the encoding of positive stimuli (Hamann and Mao, 2002). Accordingly, retrieving positive memories should also be associated with enhanced sensory recapitulation processes if those events are arousing enough to activate LC-NE systems at encoding.

Research further suggests that the degree to which positive emotional stimuli (i.e., IAPS images) engage the amygdala is modulated by arousal (Bonnet et al., 2015), and amygdala activation has been linked to emotional memory enhancements for both positive and negative arousing stimuli (Canli et al., 2000; Kensinger and Corkin, 2003). Increased amygdala activity also supports more focused encoding of both positive and negative stimuli, such that fewer peripheral contextual details are later remembered (Mickley and Kensinger, 2008). Stronger coupling between higher arousal ratings and higher emotional memory confidence have also been observed for both positive and negative valences in individuals with a NE gene variant associated with higher NE availability (Todd et al., 2013a). In a separate fMRI study, individuals with this genetic variant exhibited equivalent increases in the recall of both positive and negative photographs (de Quervain et al., 2007). While this neurogenetic work suggests a link between NE levels and the subjective quality of memory, this evidence is still indirect. Thus, more direct manipulations of LC-NE system activity (e.g., pharmacology) are needed to validate this relationship.

In sum, these data lend credence to the idea that the degree of arousal-induced NE release and amygdala activity at encoding, regardless of valence, leads to greater memory vividness and recollection during later retrieval. Thus, differences in sensory cortex reinstatement for positive and negative memoranda could emerge in a quantitative fashion (i.e., the same neural pattern but to a lesser or greater extent) given relative differences in arousal-related engagement of the LC-NE system.

### Do mechanisms beyond arousal account for differences across positive and negative memories?

Above, we described how differences in arousal across positive and negative memoranda may result in differential engagement of the LC-NE system, which leads to differences in subsequent sensory recapitulation and recollection. Yet non-arousal-related factors may also contribute to memory processing, which may explain additional variance in studies reporting valence-dependent differences in sensory cortex reinstatement.

In the following section, we outline a proposal that behavioral activation is an additional factor that shapes neural recapitulation at retrieval. In contrast to purely arousal-driven effects, we argue the behavioral activation may drive the formation of more multiplexed and integrated memory representations. Critically, previous work has only begun to explore the influence of behavioral activation on memory formation. Thus, in the following sections, we describe preliminary and supporting evidence that behavioral activation supports higher-order sensory recapitulation in areas that include more anterior portions of the ventral visual stream and those that integrate multimodal sensory information, such as the parietal cortex, medial PFC, and lateral PFC. We also provide testable hypotheses. Within this expanded framework, differences in reinstatement of sensory cortical activity patterns across positive and negative memoranda could result either from quantitative differences (i.e., how much arousal is elicited) or qualitative differences (i.e., the balance between arousal and/or behavioral activation).

### Behavioral activation as a mechanism supporting memory integration

Here, we operationalize behavioral activation as a motivational state of increased vigor and energization that promotes exploration and novelty-seeking (Alcaro et al., 2007; Düzel et al., 2010; Salamone et al., 2016). These family of related behaviors result in individuals actively exploring their surroundings, which has downstream consequences on the number of features individuals attend to in their environment. We propose that increases in behavioral activation and exploration result in a broadening of cognitive processing, thereby promoting the formation of flexible relationships between multiple features of one event (i.e., associating an item with it surrounding contextual element) and the integration of new memory traces with existing memories (i.e., updating schematic information or prior knowledge with new information). Critically, this type of mnemonic integration does not necessarily entail encoding a greater quantity of sensory details from an emotional event. In other words, this is not in direct contrast to arousal’s attention-narrowing and memory-narrowing effects towards emotional item-focused sensory features (detailed above). Rather, it specifies that enhanced associations between discrete sensory features promote the formation of higher-order cognitive representations of events.

**Figure 3.**
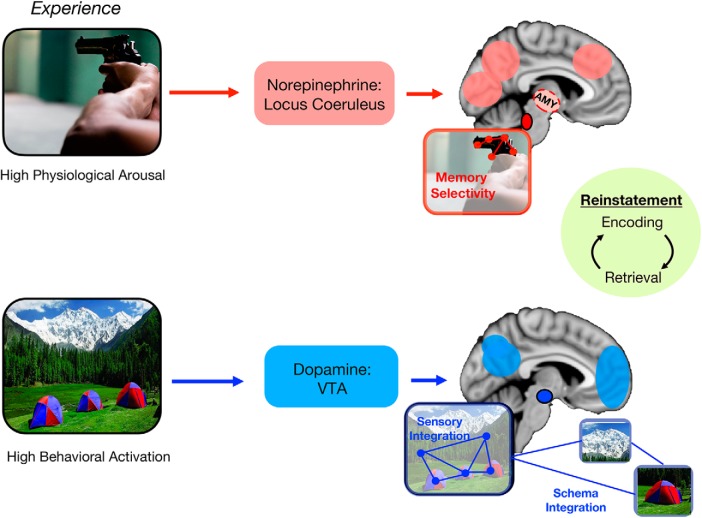
Our proposed neuromodulatory model of emotion-enhanced neural reinstatement and the quality of memory. According to this framework, high arousal levels at encoding activate the LC-NE system (red colors) regardless of valence (top panel). The LC-NE system in turn focuses limited mental resources to process high-priority (e.g., goal-relevant or perceptually salient) and/or emotional item-intrinsic representations in the ventral visual stream, amplifying encoding of a select few details. This narrowing of cognitive and memory processing under arousal is also driven by NE-related activation of the amygdala, which further modulates the selective processing of only the most salient sensory representations. This amygdala-related network may be especially important for enhancing the subjective quality of emotional memory recollection (i.e., memory confidence and vividness). Activation of the LC-NE system during post-encoding periods of consolidation will also amplify the preferential processing of salient event features even further, in part through amygdala modulation (dashed red circle; region is more lateral than depicted). By contrast, behavioral activation, defined as a motivational state of increased vigor, exploration, and novelty seeking, should recruit greater activity in the DA system that is anchored in the VTA (blue colors). In contrast to the LC-NE system, DA modulation should instead broaden the scope of cognitive processing for positive events at encoding (particularly with low arousal), leading to more integrated sensory and mnemonic representations in high-order sensory cortex and PFC. This broadening effect may be amplified by DA activation during offline consolidation and/or neural reactivation, leading to greater integration between positive memories (e.g., associative memory) and existing memory/schema in represented in PFC (blue links to other memories). In sum, engaging these different neuromodulatory systems during early stages of episodic memory should propagate downstream to enhance reinstatement processes in encoding-related sensory cortex during later retrieval (green circle). As such, which brain system becomes engaged at encoding may be the ultimate determinant of post-encoding patterns of neural reinstatement and the quality of emotional memory recollection.

To put this idea in context, imagine viewing a picture of a campsite with the backdrop of a beautiful mountain (Fig. 3). In an item-based framework, cognitive resources may be focused on encoding specific details about one of the tents in the foreground, such as its color and shape. By contrast, in an integrative memory framework, the same amount of cognitive resources may focus on the relationship between each of the tents and the surrounding forest and mountains. This experience may else invoke memories of prior camping trips. Thus, the overall amount of information may be equivalent across these encoding frameworks, but in the integrative framework, memory representations become transformed into a multi-faceted representation of discrete sensory features.

Critically, states of behavioral activation may be biased towards more positive versus negative affective states. For example, a large body of research has linked increased positive affect to behavioral activation (Custers and Aarts, 2005; Marien et al., 2013). Positive affect, particularly in relatively low-arousal states, has been shown to broaden individual’s perception, a process which enhances the global processing of events, attention to multiple features of a scene, and the integration of discrete event features (Gasper and Clore, 2002; Fredrickson, 2004; Gable and Harmon-Jones, 2008). All of these processes are intertwined with our definition of behavioral activation. For example, in an experience sampling study, spontaneous retrieval of positive thoughts was shown to predict participant’s motivation to actively engage in goal-oriented activities (Rice and Fredrickson, 2017). These types of affective states also elicit a more schematic and elaborative mode of processing and encoding (Fredrickson, 2004), perhaps due to their role in promoting self and social functions (Rasmussen and Bernsten, 2009).

Evidence from autobiographical memory studies also suggests that positive emotional experiences stimuli might in fact contain more peripheral information than negative events, at least subjectively, because memory accuracy cannot be verified in these paradigms (Bernsten, 2002; Talarico et al., 2009). In a similar vein, recent work combining an empirical manipulation with mathematical modeling suggests that positive emotion enhance associative memory for word pairs (Madan et al., 2018). What is important to note is that when experimenters attempt to match arousal levels across positive and negative valences (e.g., based on normative ratings), the arousal level is actually relatively moderate/low. This is because negative stimuli tend to be much more arousing than positive stimuli. Thus, we would expect that behavioral activation and its resulting cognitive broadening effects would prevail at relatively lower arousal states, whereas higher levels of arousal will elicit cognitive narrowing and greater memory selectivity.

While positive stimuli may be biased towards eliciting behavioral activation, it is critical to note that behavioral activation is not exclusively engaged by positive valence stimuli. Rather behavioral activation may be better aligned to the goal orientation of an individual in response to an emotional item or context. For example, studies have shown that when threats are more distant, individuals goal orientation may be directed towards active avoidance, which relies on behavioral activation and mesolimbic system (DA) engagement (Fanselow, 1994; Mobbs et al., 2009). However, when threats are more proximal to an individual, goal orientation may be directed towards defensive responses such as startle and freezing, in which circuits associated with behavioral activation are inhibited (Fanselow, 1994; Mobbs et al., 2009). One interpretation of these effects is that more proximal threats elicit greater physiological arousal, whereas lower arousal states allow the cognitive broadening effects of behavioral activation to prevail.

Our approach of characterizing behavioral activation to understand sensory reinstatement and both subjective/objective aspects of emotional memories parallels several theories of emotion and motivation. For instance, our ideas dovetail with the evidence suggesting that goal orientation may modulate the scope of attention and memory processing (Gable and Harmon-Jones, 2011; Harmon-Jones et al., 2013; Kaplan, 2013), with pre-goal attainment states and emotions, such as desire or anger, being associated with cognitive narrowing effects, and post-goal attainment states and emotions, such as happiness or sadness, being associated with cognitive broadening (Harmon-Jones et al., 2013; Kaplan, 2013). Thus, beyond valence, goal orientation may be a key factor for determining the breadth of information that is processed and encoded into long-term memory. High-arousal states have also been likened to the effects of “motivational intensity,” or the strength of approach or avoidance emotions, on cognitive narrowing effects (Harmon-Jones et al., 2013). These arousal-like effects on memory are also invariant to emotional valence.

Similarities notwithstanding, the role of goals and emotional states/mood in cognitive processing is somewhat challenging to reconcile with other emotion-cognition studies of declarative memory. Namely, the former class of studies are generally designed to modulate an individual’s subjective emotional experience (e.g., sadness, happiness, anger). As such, they might not map clearly onto most of the emotion-cognition memory studies we have reviewed, which tend to query emotional memory for discrete emotional words, sounds, or pictures (for a short discussion, see Harmon-Jones, 2018).

### Behavioral activation may drive high-order patterns of neural reinstatement

The majority of studies characterizing behavioral activation’s influence on cognition have focused on early stages of processing, including perception, attention, and encoding. However, these studies lay a strong foundation for making predictions about how behavioral activation may influence memory reinstatement patterns both after encoding and during retrieval. We propose that sensory cortex reinstatement, in the context of behavioral activation, would likely emerge in higher-order brain processing regions, including multi-model sensory cortex and PFC. These neural patterns might therefore reflect high-order cognitive representations that link together multiple elements of sensory events. Although this facet of emotional memory has yet to be tested directly, positive emotional stimuli which tend to be both lower in arousal but also elicit behavioral activation, have been more closely linked to engagement of higher-order sensory cortex, such as more anterior portions of the ventral visual stream (e.g., fusiform gyrus and perirhinal/parahippocampal cortex), as well as PFC regions associated with memory integration and semantic elaboration, such as ventrolateral PFC, orbitofrontal cortex, and medial PFC (Blumenfeld and Ranganath, 2007; Badre, 2008; Preston and Eichenbaum, 2013).

The above framework might help explain why there are some discrepant findings concerning whether patterns of sensory cortex reinstatement are similar across positive and negative valences after encoding. Specifically, these inconsistencies could result from greater variability in the amount of behavioral activation evoked by memoranda. For example, a picture of a smiling face may evoke behavioral activation, activating neurocognitive systems known to facilitate exploration and vigor (Mende-Siedlecki et al., 2013). By contrast, other positive stimuli, such as a picture of a sunset, may induce positive affect in the absence of promoting behavioral activation. Viewed through this lens, emotional stimuli could be varied in their content in a manner that induces qualitatively different types of neural reinstatement, sometimes promoting memory integration while other times promoting selectivity. However, to explore these ideas, future studies need to begin to quantify the extent to which memoranda evoke behavioral activation (see below, Objective measures of arousal, behavioral activation, and neuromodulation).

One way to gain insight into mechanisms by which behavioral activation evokes memory reinstatement is to investigate memory enhancements in response to reward anticipation. Reward anticipation, particularly in the absence of feedback, may serve as a more reliable stimulus to induce behavioral activation than those induced by positive emotional stimuli (e.g., positive IAPS images). Reward anticipation has been shown to increase recollection and source memory during memory retrieval, processes that are thought to rely on enriched retrieval of sensory details from encoding (Wittmann et al., 2005; Adcock et al., 2006; Shigemune et al., 2014). fMRI research in humans has also identified greater post-encoding reactivation of sensory stimulus information encoded during reward anticipation (Murty et al., 2017), as well as increased engagement of higher-order sensory and prefrontal cortices by rewarding memoranda during retrieval (Elward et al., 2015). Sensory recapitulation of information encoded during reward anticipation may involve more integrative representations of sensory information (Murty and Adcock, 2017), which would be represented in higher-order sensory and multi-sensory cortex (Schlichting and Preston, 2016).

Notably, these states of reward anticipation detailed above differ from studies which utilize instrumental learning paradigms with performance feedback. In these contexts, individuals may narrow their attention towards the instrumental action necessary to receive positive feedback, thus inhibiting behavioral activation. For example, a recent study characterized how reward influenced memory in the context of a paradigm in which individuals made instrumental actions to receive reward feedback (Gable and Harmon-Jones, 2010). This study found a narrowing of memory and attention during the “pre-goal” phase, i.e., after the reward cue but prior to the instrumental action. Interestingly, this study also found that successfully receiving rewards resulted in “post-goal” emotions which broadened cognitive processing.

### The balance between behavioral activation and arousal shapes the scope of memory

**Figure 4.**
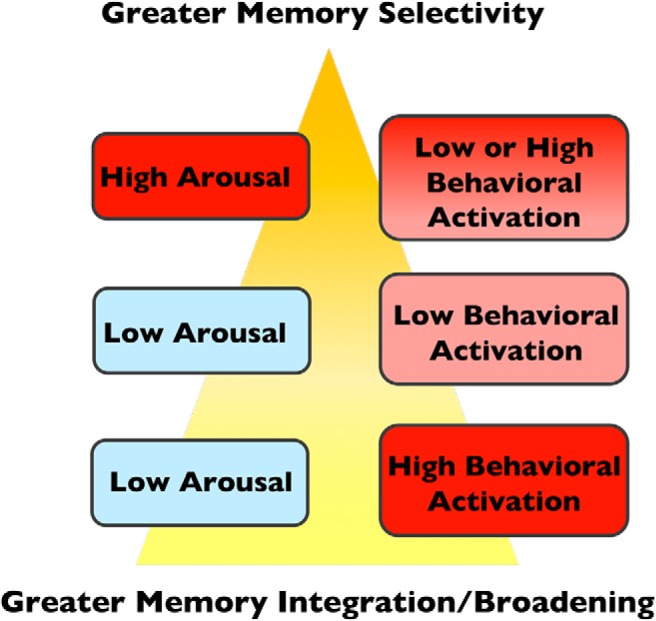
Putative interactions between arousal and behavioral activation, and their effects on the scope of cognitive and memory processing. According to our framework, there may be interactions between arousal and behavioral activation that influence the balance between memory selectivity versus memory integration. We predict that high-arousal states will elicit greater memory selectivity, regardless of the level of behavioral activation (top row). By contrast, under states of high behavioral activation and low arousal, we predict that there will be greater memory integration and/or broadening (bottom row). These broadening versus narrowing effects occur relative to states of low arousal and low behavioral activation, which we would also characterize as neutral (middle row).

So far, we have detailed how factors associated with increased behavioral activation may elicit a different form of sensory cortex reinstatement than arousal. However, critical questions remain about how these two constructs interact. In our view, arousal and behavioral activation are typically orthogonal constructs, but not in all cases. Low levels of arousal but high levels behavioral activation will lead to cognitive and memory broadening. But high arousal will tend to supersede the effects of behavioral activation (or in other words, be invariant to behavioral activation levels), thereby leading to cognitive and memory narrowing. Neutral circumstances, on the other hand, represent states of low behavioral activation and low arousal (Fig. 4).

To illustrate these ideas, consider the following scenarios. Encountering a threatening snake in a zoo may elicit high arousal but not necessarily high behavioral activation, as the threat is confined to a cage; this would theoretically narrow the scope of attention and memory to emotional items, including the snake. By contrast, seeing a threatening snake at a distance on a hike may evoke both high behavioral activation and moderate levels of arousal, such that individuals may explore their environment for escape options; this in turn broadens the scope of attention and memory to process and integrate multiple features and associations in memory. Almost stepping on a snake during a hike, however, may evoke such a level of sympathetic arousal that behavioral activation processes become inhibited. Indeed, this type of behavioral suppression is often observed with threat-induced freezing behavior in both rodents and humans (Fanselow, 1994; Ly et al., 2014).

In line with this example, recent research has shown that the proximity of a threat can modulate defensive responses, which are akin to freezing behavior, suggesting that arousal responses to negative valence stimuli are modulated by other contextual features (Åhs et al., 2015). Similarly, in the domain of positive affect, early exposure to drugs of abuse in rodent models elicit greater behavioral activation, as assayed by exploratory behavior and vigor (Alcaro et al., 2007; Alcaro and Panksepp, 2011); but notably this body of research does not explicitly address arousal. However, in cases where drug cues are extremely salient and induce intense levels of stress and arousal, such as in the context of craving and/or relapse, behaviors may become more habitual and behavioral repertoires become selective only to actions that may elicit acquiring more drugs (Sinha et al., 2003; Sinha, 2013). In line with these interpretations, recent human research has shown that when reward motivation elicits highly arousal states integrative memory can be disrupted, and memory representations can be biased towards cues that directly predict reward at the expense of surrounding contextual details.

From a neuromodulatory point of view, NE levels have also been shown to be elevated during craving and acute withdrawal (Fitzgerald, 2013), and β-adrenoreceptor activation may facilitate a shift from goal-oriented towards habitual responding under stressful conditions (Schwabe et al., 2011). Together, these studies suggest that high arousal will narrow the scope of attention and memory towards salient information regardless of the level of behavioral activation, perhaps in part by inhibiting exploratory behaviors.

### Does neuromodulation explain the putative effects of behavioral activation on neural reinstatement?

The nature of neural and memory reinstatement patterns evoked by behavioral activation likely involves the engagement of brain systems beyond the LC-NE system. One such candidate mechanism is the mesolimbic DA system. The DA system, which is anchored in the DA substantia nigra and ventral tegmental area (SN/VTA), has been linked to aspects of behavioral activation (Alcaro et al., 2007; Alcaro and Panksepp, 2011). For instance, DA system activation occurs under states of reward motivation that are critical for translating individuals’ goals into approach-related actions (Berridge and Robinson, 2003; Wise, 2004). While there is less evidence supporting the mesolimbic DA system’s role in memory recollection, we will speculate on this possibility based on evidence that DA promotes associative memory formation.

Accumulated evidence suggests that DA activity contributes to increased sensory processing (Noudoost and Moore, 2011) as well as increased memory reactivation/recapitulation (Wang and Morris, 2010). Further, the DA system may be driving sensory processing and recapitulation in a different way than the NE system. DA receptors are found throughout the brain, but in very low densities in primary sensory cortical regions; DA axonal projections to primary sensory cortex are relatively sparse, especially compared to LC neurons (Jacob and Nienborg, 2018). SN/VTA neurons send dense neuronal projections to higher-order sensory processing regions in anterior portions of the ventral visual stream, which are known to support the relational binding between the elements of experience (Davachi, 2006; Graham et al., 2010). VTA neurons also heavily innervate lateral and medial PFC (Robbins and Arnsten, 2009; Jacob and Nienborg, 2018), which could facilitate more elaborative, self-referential processing and support the integration of incoming information with existing overlapping memories (van Kesteren et al., 2012). Consistent with this possibility, research in rodents and non-human primates show that increased DA signaling can influence prefrontal-mediated mechanisms of attention and downstream engagement of sensory cortex at a higher-order level of processing (Noudoost and Moore, 2011; Jacob and Nienborg, 2018).

This functional neuroanatomy of the DA system makes it well positioned to facilitate the integration of multimodal sensory information under high states of behavioral activation. Research has shown that engagement of the DA system during encoding leads to more integrative memory representations (Shohamy and Wagner, 2008). SN/VTA activation parallels hippocampal activation during the anticipation of novel stimuli, which has been interpreted as the DA system driving the exploration of new environments (Wittmann et al., 2007). Furthermore, recollection is enhanced for anticipated novel stimuli, suggesting that DA modulation may drive exploratory behaviors that promote encoding of salient, novel events (Wittmann et al., 2007).

There is also fMRI evidence to suggest that DA modulation is important for facilitating associative memory binding during consolidation. Greater post-encoding interactions between the VTA, a central hub of the DA system, and higher-order sensory cortex, such as the perirhinal cortex and category-selective visual cortex, relates to enhanced associative memory (Tompary et al., 2015; Murty et al., 2017, 2018). Although these post-encoding fMRI measures do not directly assess brain activity at retrieval, it has been posited that post-encoding reactivation during rest involves similar mechanisms to sensory reinstatement or reactivation at retrieval (Antony et al., 2017).

How memoranda are encoded and consolidated has important consequences for how those experiences are later recapitulated and remembered. In contrast to the attention and memory-narrowing effects of NE under arousal (Mather et al., 2016), emotion-related engagement of the DA system may broaden the scope of cognitive processing during the encoding of rewarding or positive events (Ashby et al., 1999). As described above, however, we would attribute these effects to behavioral activation rather than positive emotion, per se. Insofar as encoding processes dictate which sensory information gets stored and later reactivated at retrieval, this more generalized form of cognitive processing may enhance the associative relationships among different elements of an event (Duncan and Schlichting, 2018). Indeed, at the neural level, activation of the DA system has been shown to enhance associative memory binding and memory generalization both in the context of positive (Wittmann et al., 2005; Murty et al., 2011; Wolosin et al., 2012; Shigemune et al., 2014) and negative events (Clewett et al., 2018b).

Critically, more work is needed to determine the consequences of engaging both arousal and behavioral activation to understand the complex interactions between these neuromodulatory systems during the encoding of motivationally significant events. For example, highly arousing positive stimuli, such as drug cues, could engage both DA and NE systems, given their propensity to evoke both behavioral activation and arousal (Sinha et al., 2003; Koob and Le Moal, 2005; Economidou et al., 2011). Preliminary data suggest that more selective encoding versus broader, higher-order representational encoding will prevail whenever arousal levels are sufficiently elevated (Callan and Schweighofer, 2008; Murty et al., 2011). However, this idea has yet to be tested explicitly, particularly within the context of human emotional memory. An important direction for future research is to explore how these brain systems either work in concert or counter to one another to modulate the quality of emotional memory recollection and corresponding patterns of neural reinstatement in sensory cortex and across the brain.

### Testing the factors that drive emotion-related neural reinstatement and memory recollection

In many ways, the neuromodulatory model we have proposed is preliminary. While grounded in strong theoretical work, more empirical research is needed to test our specific predictions and to compare these predictions against those put forth in the NEVER model. Below we detail some methodological approaches to testing these frameworks.

### Controlling differences in arousal and behavioral activation across valences.

Acquiring participant-specific arousal, valence, as well as behavioral activation assayed through self-report ratings (e.g., BIS/BAS; Carver and White, 1994) as well as behavioral measures (i.e., response vigor) is essential, as there is substantial variability in these ratings across individuals. Some ways to also account for such variability is to integrate item-specific ratings into trial-level linear mixed models, which would allow for direct comparisons of valence versus arousal/behavioral activation effects.

### Objective measures of arousal, behavioral activation, and neuromodulation.

In addition to self-report measures, it will be critical to also assay objective measures of arousal and behavioral activation, as well as their relationship to neuromodulatory system activity. An especially effective way of assessing the effects of arousal on memory is to use more objective physiological measures, including eye-tracking and skin conductance responses. Increasing research in both humans and animals suggests that pupil dilation may be a reliable index of LC activity (Murphy et al., 2014; Varazzani et al., 2015; Joshi et al., 2016; Clewett et al., 2018a). Human research has begun to utilize tools to characterize behavioral activation, including characterizing how stimuli invoke vigor, through paradigms using finger tapping (Heerey and Gold, 2007), exploration of visual scenes using eye-tracking (Voss et al., 2017), and the ability of stimuli to evoke approach behaviors using Pavlovian-to-Instrumental transfer paradigms (Talmi et al., 2008). Further, research has begun to show that measures of blink rate may index DA activity both at rest and during cognitive tasks (Jongkees and Colzato, 2016). fMRI measures could also be used to measure BOLD signal changes in the LC and VTA/SN during the encoding, consolidation, and retrieval of emotional memories. Finally, up-regulating or down-regulating activity in the NE and DA systems using pharmacological manipulations would also provide evidence of more causal relationships between neuromodulation and emotional memory recapitulation.

### Measuring the selectivity of emotional memory recollection.

One of the key questions we have raised concerns the types of information that are being recapitulated during emotional memory retrieval. To address this issue, future research should carefully manipulate which information is prioritized in attention and memory under emotional circumstances (for discussion, see Mather et al., 2016), and query how memory for high and low priority information relates to sensory recapitulation patterns at retrieval. Similar specificity could be achieved by using more sensitive measures of neural recapitulation with fMRI data, such as multivoxel pattern similarity analyses. This approach targets the precise correspondence between individual item representations when they are initially encoded and later retrieved (Ritchey et al., 2013). Recent work also shows that similarity in eye movements and fixation patterns across encoding and retrieval may provide an index of item-specific reactivation (Johansson et al., 2012). Interestingly, research also suggests that using picture-drawing to probe the recall of previously studied scene images might also help to reveal the mnemonic content of those experiences (Bainbridge et al., 2019). Using this type of memory probe for emotional scenes could help reveal the types of perceptual information that people encode.

### Manipulating study-test retention intervals.

Research suggests that retention interval may play an important role in modulating recapitulation effects, given that emotion typically has a more pronounced effect on recollection over time (Sharot et al., 2007, 2008; Ritchey et al., 2008; Yonelinas and Ritchey, 2015). Of interest, behavioral work suggests that valence effects on memory may interact with delay and therefore impact the magnitude and/or nature of recapitulation (Pierce and Kensinger, 2011). This facet of recapitulation would be especially relevant, as both the LC-NE and DA systems enhance emotional memory during consolidation (McGaugh, 2004; Shohamy and Adcock, 2010).

### Summary of our neuromodulatory model of emotional memory reinstatement

Our most vivid and enduring memories tend to be emotional. The specific factors and neural processes that drive such detail-laden recollections, however, are somewhat unclear. The recently proposed NEVER model provides an interesting account of this phenomenon by targeting how emotional memory traces laid down at encoding shape the information that is available for later reinstatement. It is posited that, by re-engaging the same perceptual processing mechanisms that operated at encoding, retrieval quite literally reflects the re-experience of prior events. While this model focuses primarily on the role of negative valence in driving this encoding-retrieval overlap, we argue that the bulk of evidence still favors arousal as the key factor driving enhanced emotional memory recollection and vividness. We also provide preliminary evidence to investigate behavioral activation factors as an additional mechanism contributing to the quality of recollection.

At the crux of our model is that arousal and behavioral activation, as well as the neuromodulatory systems they activate, may be better predictors than valence of neural reinstatement patterns and the quality of retrieved emotional memories. From the perspective that encoding and neuromodulatory processes determine the details that are stored in memory, we argue that sensory and high-order cortex reactivation after learning reflects biases in which features of events are reflected in memory (Fig. 3). That is, patterns of reactivation may reflect either a more selective memory representation or something more abstract and generalized.

In our model, an increase in arousal and LC-NE activity promotes the prioritization of the most salient features of an emotional experience at encoding, whereas more mundane sensory information may be initially neglected, and later distorted and/or forgotten. Thus, an inflated sense of confidence and memory vividness for emotional events may be driven by especially strong representations of select details of those events. At present, much research suggests that amygdala may act independently of or in concert with NE activity to amplify these effects across each stage of episodic memory.

In parallel to these encoding mechanisms driven by arousal-related LC-NE system activity, reinstatement effects in higher-order cortex may be driven by behavioral activation and, possibly, activation of the mesolimbic DA system during encoding and post-encoding rest. This form of DA modulation, however, may be qualitatively different from LC-NE system modulation. Under states of elevated DA system engagement, including exploration, novelty, and behavioral vigor, sensory features of the environment may become transformed into more integrated, higher-order representations of events. Positive emotions often co-occur with this class of behavioral responses, which might explain why they also tend to elicit cognitive and memory broadening effects, at least under states of lower arousal or motivational intensity (Harmon-Jones et al., 2013). Further, this integrative mode of processing may promote integration between information in the environment and existing memories or schema represented in the PFC.

In sum, the goal of our framework is to move beyond the idea that valence drives differences in reinstatement. Rather, we propose that the degree of arousal and behavioral activation during learning influences downstream patterns of neural reinstatement that manifest qualitative differences in memory (Fig. 4). To reconcile our proposal with valence-related models like NEVER, we propose that negative stimuli may be more likely to evoke higher arousal levels than positive stimuli, and thus could yield greater neural reinstatement in relatively lower-level sensory cortical regions and the amygdala. Conversely, positive stimuli may be more prone to evoke a sense of behavioral activation than negative stimuli, as negative images can often instead promote behavioral inhibition and freezing responses (Azevedo et al., 2005; Blakemore et al., 2016). While speculative, positive events might therefore be more likely to activate the DA system at encoding and consolidation (Murty and Adcock, 2017), leading to better associative memory.

Careful experimental manipulations of the relative priority of multiple features of an experience and an individual’s memory for their associations could help unpack the brain and behavioral factors that impact both subjective and objective aspects of emotional memory. To test these predictions, for example, future emotional memory studies would need to systematically vary behavioral activation in addition to arousal across valences, and measure the magnitude and spatial patterns of neural reinstatement following encoding. This could be achieved by modifying traditional emotional memory studies to manipulate exploratory goal states and to quantify the extent to which individual memoranda increase vigor/energization, and/or to manipulate the relative novelty of memoranda. Using multivoxel pattern similarity analyses may be a particularly effective way of quantifying encoding-retrieval overlap for both trial-unique and category-level effects (Ritchey et al., 2013).

## Conclusion

Characterizing the influence of neuromodulators and emotion on recollection has important implications for cognition in healthy individuals and individuals with disorders of emotion. Age-related changes in neuromodulatory systems may have a profound impact on how emotional memories are encoded, stored, and later recollected. These differences may be particularly pronounced in age-related neurodegenerative diseases, such as Alzheimer’s and Parkinson’s disease, in which decline in the NE and DA systems is significant (Vazey and Aston-Jones, 2012; Robertson, 2013; Mather and Harley, 2016).

The work we have reviewed also has relevance concerning the recapitulation of more naturalistic, real-world memories. In particular, extending this research into the domain of autobiographical memory has clinical relevance to disorders characterized by maladaptive episodic memory binding, such as post-traumatic stress disorder (PTSD). More recently, it has been shown that PTSD is associated with hyper-responsivity of the LC under arousal (Naegeli et al., 2018), suggesting that treatments targeting NE activity could be an effective way of weakening aversive responses and maladaptive memories (Kroes et al., 2016).

Emotions are multifaceted, and there are undoubtedly more complex interactions between arousal, motivation, and cognition, as well as between these neuromodulatory systems, than our preliminary model can address. But we hope that our ideas will invigorate new discussion about the nature of emotional memory and its underlying mechanisms. Through our framework, we encourage cognitive neuroscientists to broaden their focus to include neuromodulation when examining emotion’s persistent effects through each stage of episodic memory. Doing so may better inform how bringing different emotional memories to mind impacts how we learn and maintain a sense of wellbeing in the present.

## Synthesis

Reviewing Editor: Morgan Barense, University of Toronto Decisions are customarily a result of the Reviewing Editor and the peer reviewers coming together and discussing their recommendations until a consensus is reached. When revisions are invited, a fact-based synthesis statement explaining their decision and outlining what is needed to prepare a revision will be listed below. The following reviewer(s) agreed to reveal their identity: Daniela Palombo.

Synthesis:

1. Broadly speaking, both reviewers agree that this paper has the potential to be an important contribution that will generate needed progress in this field, especially because many of the predictions have yet to be tested. This discussion is also timely because we have many new exciting methodologies for examining recapitulation effects that will nicely build on the existing approaches used in other work. Still, both reviewers agreed that the new paper left much unsaid. For example, the authors need to flesh out their predictions and offer stronger conclusions. Perhaps, this could be in the context of unpacking future directions -what needs to be tested, what are the appropriate methods, etc. In the context of this point, it was noted that the current review does not do justice to the NEVER model (the description is piecemeal) and as a result some of the important nuances of the NEVER model are overlooked.

2. Both reviewers commented about whether we can draw on the encoding and memory literature in isolation to shed light on whether arousal actually plays a role in the negative specific effects of recapitulation observed in some papers. Reviewer 2 also stated that if it were just arousal, you might expect differences between positive and negative (at encoding or retrieval) to be quantitative not qualitative, and that is not what one sees in the literature. For example, Steinmetz et al showed that when arousal levels were equated, negative and positive valence showed opposite neural patterns rather than similar patterns - suggesting valence specific effects of arousal. Perhaps, it is at a certain level of arousal that valence patterns in sensory processing begin to dissociate (of course these needs further testing).

3. Both reviewers agreed that the utility of reward as a surrogate for positive emotion is overstated. It is acceptable to make this comparison, but it is necessary to emphasize that there are differences between reward and positive emotion. In practical terms, there were doubts regarding whether this surrogacy could work (e.g., Bowen & Spaniol, 2017).

4. Both reviewers were confused about what one can expect for positive events in this new model. As articulated by Reviewer 2: “Broadening of attentional scope at encoding when the positive stimulus is presented, should lead to better memory for the positive stimulus and background/context. Theoretically this broadening would lead to more sensory input and therefore more sensory engagement at encoding that is then recapitulated at retrieval.” This seems at odds with the authors' statement that “...emotion-related engagement of the DA system is associated with broadening the scope of attention during the encoding of rewarding or positive events (Ashby et al., 1999). Insofar as attention dictates which sensory information gets stored and later reactivated at retrieval, this more generalized form of sensory processing may facilitate the retrieval of flexible memory representations and promote the integration of new emotional information with existing memories”. There was confusion regarding what this means in terms of neural and behavioral predictions. Is it that there is a lot of sensory activation at encoding for positive events due to the broadening of attention, but that there is less so at retrieval due to post-encoding transformation of the mnemonic representation to a more schematized/integrated form (leading to low overlap)? Or perhaps the authors meant something else. Regardless, this illustrates why it is so important to couch recapitulation effects against what happens at encoding and retrieval in isolation.

5. We agreed that it would be helpful to unpack in more detail the comparison to the Ritchey paper, and how they relate to the ‘NEVER’ model. The authors suggest the Ritchey paper is against the NEVER model, however the findings regarding similarity-amygdala correlation in the service of successful memory for negative but not positive are actually in line with the NEVER model.

6. The two reviewers diverged in terms of how they viewed this new model. Reviewer 1 noted that because the authors are disputing one prong of the NEVER model, their point of view seemed to be a challenge to the NEVER model. In contrast, Reviewer 2 sees the new model as complementary. This reviewer stated: “I think there can be influence of arousal, activation of DA and NE systems, and valence differences in sensory focused processing, but I also think we are a long way from understanding how these systems might interact. As pointed out, a more detailed description of DA and NE effects on broadening and narrowing, might be fruitful for making predictions about how these systems might work together (or perhaps compete).” Please consider these comments and the positioning of your model with respect to NEVER in your revision.

Reviewer 1:

In their review, the authors build on (and in some places challenge) ideas put forth by an interesting and provocative new model (i.e., the NEVER model; “Negative Emotional Valence Enhances Recapitulation”), which argues that emotional memory enhancement effects, particularly for sensory details are associated with (or facilitated by) greater sensory-based recapitulation effects (measured as encoding-to-retrieval overlap) than neutral ones, and that this is most prominent for negative memories. A review of this nature is timely and important and the authors emphasize the importance of recapitulation effects that are associated with positive memories. This undoubtedly will spur interesting discussion in the field. However, there are some concerns I have that dampen my enthusiasm for the paper in its current form.

Major:

Discussion of NEVER: Before turning to the specifics of my review, it is worth mentioning that although the authors nicely bring together many branches of the literature, the description of the NEVER model and the associated literature is peace meal. Considering that the NEVER model is based, to a large extent, on the work of Kark et al., and the Bowen et al. papers, it would be helpful if the authors unpacked those index papers more: (1) What were the behavioral effects in those studies: Is there evidence from those papers that sensory memory (or any memory) is augmented for negative as compared to positive memories? (2) Precisely where are these recapitulation effects observed in those datasets? The authors state: “The NEVER model proposes that the sensory features of a negative emotional event are reactivated in sensory cortex during retrieval.” But they do not actually state whether this is true in those data sets and precisely whether these recapitulation effects occur outside of sensory cortex as well. This is notable because they later speak about the amygdala findings from other papers but it is my understanding that the amygdala findings in the index Kensinger papers were more nuanced. The reader needs this basic background information. There are several instances in the paper where this is an issue and I find the discussion of amygdala findings in particular a bit misleading/superficial. (3) My understanding is that the NEVER model makes a lot of predictions, some that are supported by existing data, and some that have not yet been tested. Can the authors briefly clarify this?

Emphasis on positive events: The authors' emphasis on the importance of recapitulation effects for positive memories and the issue of arousal versus valence confounds is important. The authors further argue that there may be different encoding-based neuromodulatory mechanisms for negative versus positive memories, that lead to differences in the degree of narrowing versus broadening of attention and they cite supporting literature showing that negative and positive memories indeed diverge to some degree in their neural and neuromodulatory mechanisms. At a more cognitive level, they argue that narrowing may lead to a honing in on sensory details, whereas broadening might lead to more elaboration or schematic processing (and even subsequent generalization to other experiences). Thus both types of memories would involve mnemonic enhancements but the mnemonic representations would be qualitatively different. While I like the spirit of this idea, I think these ideas need further unpacking. What exactly would the mnemonic predictions be in a garden-variety emotional memory paradigm for negative and positive items like the one used in Kark? The prediction for negative events is straightforward, but what would the mnemonic representation ‘look like’ for positive items under this ‘schematized’ view? Moreover, what would this actually mean with respect to the neural correlates of recapitulation for negative versus positive experiences? Would they be quantitatively similar but qualitatively distinct? Perhaps the Ritchey work that the authors cite is relevant here. Can the authors unpack this a bit better.

What is the best way to measure ‘recapitulation’? The Kensinger series of papers uses encoding-to-retrieval overlap (using conjunction approaches) as their putative measure of recapitulation. Generally speaking, there are some caveats to this approach. For example, one has to be sure that greater recapitulation for e.g., negative versus positive is not just driven by greater encoding (or retrieval) effects for negative per se (to use an extreme example, if the entire brain is active during encoding of negative events, even if only a small area was active for retrieval, the overlap would be high. The authors deal with this to some extent, with control analyses, but it makes me wonder if there are other approaches, such as representational similarity analyses or overlap metrices (that control for non-shared overlap). Along these lines, it could be worth mentioning at some point whether there are any pertinent methodological/behavioral differences that are relevant between paradigms that do and do not find similar recapitulation effects for negative versus positive events (Kensinger studies versus e.g., Ritchey work). More broadly, can we learn something about how to understand these recapitulation effects by couching them in terms of what happens at encoding and retrieval in isolation? For example would examining encoding in isolation help clarify (albeit speculatively) whether arousal indeed mediates greater recapitulation effects for negative versus positive events?

Clarification of terms: As noted below, there are several places in the paper where the authors are a bit unclear/imprecise in their use of terms.

on p.8. the authors imply that post-encoding activation is a “putative marker or recapitulation.” Is this really true?

on p.7, the authors refer to novel events as positive ones. I think that is painting novelty with an overly specific brush.

The authors suggest that reward can serve as a surrogate for positive to sidestep arousal issues. I liked the spirit of the idea, especially because, in my view, there is not enough cross talk between the emotional memory and value memory literatures. However, how far should we take this? Do we think the neural mechanisms of reward and positive emotion are similar enough to warrant this approach in practice? For example, do we think positive experiences lead to the same types of prediction errors as do rewarding stimuli? Do you think the same surrogacy can apply to negative stimuli? To be extreme in illustrating this point, why not then just study reward and punishment, since it allows for better matching between stimuli? More broadly though, spread of value effects occur for both punishing and rewarding stimuli (i.e., the generalization literature)-how does this fit with the notion laid out by the authors about narrowing versus broadening (and subsequent schematizing) for negative versus positive stimuli, respectively. I am having trouble parsing these ideas.

On the topics of surrogates, I wondered also about a consideration or short discussion about the relevance of this work to more naturalistic mnemonic processes (i.e., autobiographical memories). By definition, we cannot test recapitulation for autobiographical memories. But do we think that laboratory stimuli are a good placeholder for ultimately understanding real-world memory (this might be important if this work is going to inform clinical effects, such as those observed in PTSD). This may be beyond the scope of this paper but it might capture the attention of a more diverse audience.

The authors state: “Supporting this idea, human memory studies utilizing pharmacological and genetic approaches have shown that putative increases in NE tone at encoding enhances the subjective recollection and vividness of emotional memory (Rimmele et al. 2016), in part through increased amygdala influence over sensory cortex (Todd et al., 2013a, 2015).” The authors should temper this statement as the genetic work is indirect and some of those data are correlational. Moreover, one of the Todd et al. papers involves direct examination of neural mechanisms (the J Neuroscience paper).

The authors argue that their model is complementary. In one way, this is true, as they concur with the authors that emotional memories involve recapitulation of sensory information. However, the main crux of the NEVER Model is that the putative recapitulation effects are specific to negative memories. This feels more of a challenge to the NEVER model than a complementary idea per se.

Other:

Do the authors have any thoughts about retention intervals? We know that the effects of emotional memory are stronger after a delay. Are the laboratory studies that have examined recapitulation effects adequate for addressing issues of delay with respect to valence effects? One might (or might not) predict that recapitulation effects for negative versus positive events interact with delay. I think this is worth mentioning, i.e., when we are testing predictions generated by the NEVER model, we need to consider the delay.

Minor:

Page 4: neutral has an extra ‘u’

The 2nd paragraph on page 3 is a bit disorganized. The authors start by briefly mentioning the R K paradigm (which involved subjective reporting) and then turn to studies of source memory and then turn back to subjective reporting (confidence, vividness etc). Can the authors organize this better?

Reviewer 2:

This is a well-written perspective on how activity in neuromodulatory arousal systems, particularly the noradrenergic and dopaminergic systems, can influence emotional memory. The paper is written in contrast to the recently published NEVER model, which emphasizes valence effects on sensory recapitulation and memory advantages for negative events.

I appreciate the points made in the paper, and conceive of this account as being complementary to the NEVER model, as the author(s) suggest. It is possible that the mechanisms proposed in both papers are interacting to support emotional memory overall, but could also support valence differences in memory. Perhaps what is not explicitly stated in the NEVER paper is that evidence for valence effects may really be valence specific effects of arousal. Although there is evidence that negative and positive stimuli sometimes engage or recapitulate sensory cortices to the same extent, it seems that only for negative stimuli is this in the service of memory. I will comment on this and a few other points more explicitly below.

1. One of the first points in the paper is that salient information coupled with arousal will result in vivid recollection. Further, negative stimuli are often more salient or arousing than positive or neutral and this confound can explain many of the valence effects found in memory. I am having difficulty piecing together a few points in your paper related to this argument, particularly how salience or arousal can account for valence differences in sensory cortices associated with a neutral retrieval cue. I'll try to break up my thoughts for clarity.

a. Much of the evidence in the NEVER model comes from studies where memory retrieval is cued by neutral memoranda that were previously (often unintentionally) encoded with an emotional stimulus (e.g., Bowen & Kensinger, 2017). If negative stimuli are more salient, more arousing and engage the NE system, you suggest this will lead to a narrowing of attention at the expense of information in the periphery. It follows then, that memory for peripheral information and the corresponding retrieval cue would be impaired. Further, when the negative memory is being cued by a neutral stimulus that provides no information about the prior encoding context or emotion, this should result in less visual or sensory activity at the time of retrieval since the prioritized information from encoding would have led to inattention to this part of the stimulus. But this logic is in contrast to the evidence presented in the NEVER paper which finds more sensory activation and recapitulation during successful memory of these neutral retrieval cues previously encoded with a negative stimulus. This perhaps suggests that the negative stimuli were not in fact more salient or arousing than positive.

b. The opposite should be true for positive stimuli. Near the end of the paper you discuss research on how positive emotion leads to activation of the dopamine system and a broadening of attention. Broadening of attentional scope at encoding when the positive stimulus is presented, should lead to better memory for the positive stimulus and background/context. Theoretically this broadening would lead to more sensory input and therefore more sensory engagement at encoding that is then recapitulated at retrieval, but according to the NEVER paper, sensory (re)activation happens to a lesser extent than negative and even less than for neutral stimuli.

c. It seems that in contrast to statements in the current paper, valence differences in memory and sensory engagement/recapitulation cannot be totally accounted for by stimulus confounds between the valences (e.g., Kark & Kensinger, 2015 differences in arousal), particularly when the retrieval cues are devoid of emotion and provide very little sensory input.

2. If confounds such as arousal, salience or visual complexity could really account for all the valence differences in memory, I would think that the activation and connectivity patterns would be similar between the two valences, but with positive showing the pattern to a lesser degree than negative; however, this is not what most report and some even report opposite effects for negative and positive valence. For example, Mickley Steinmetz, Addis & Kensinger, 2010 in Neuroimage, matched positive and negative valence on a number of factors including arousal, visual complexity and content category. They found opposite patterns for negative and positive valence, such that there were stronger amygdalar efferents to sensory regions for high compared to low arousal negative items, but the opposite pattern for positive. For positive items, low arousing items resulted in stronger amydalar connectivity to sensory regions. In other words, high arousal positive items were associated with decreased connectivity between amygdala and sensory regions whereas high arousal negative an increase. These findings suggest that arousal and valence are interacting, but the modulation of arousal on valence is in opposite directions. How does this fit with your idea of an arousal/salience account of emotional memory?

3. You discuss positive valence in the form of reward, providing some research showing that positive valence defined as reward motivation is associated with increased recollection, source memory and reactivation in sensory cortices. Although this may be true, I don't think this is a strong argument against negative emotion modulating memory in a different way than positive emotion. It is well established that reward motivation and positive emotion are not the same. One is considered top-down and the other bottom-up, they rely on different neurotransmitters, overlapping but separate brain regions, and do not have equivalent effects on cognition (e.g., Chiew & Braver, 2014, Berridge & Aldridge, 2008, Gable & Harmon-Jones, 2010, Emotion, Fröber & Dreisbach, 2014). Reward motivation having similar effects on memory processes as negative emotion does not negate valence differences when comparing negative and positive emotion in the way that is described in the NEVER model.

4. In this same section you cite Ritchey et al., 2013, highlighting their finding that encoding-retrieval similarity was equivalent for positive and negative stimuli in sensory cortices. This is true when they were assessing activation during perception at encoding and perception of that same item at retreival, however when they were looking at whether encoding-retreival similarity modulates memory, they find that in fact there are valence difference associated with sensory cortex. The encoding-retreival similairty in middle occipital gyrus correlated across trials with activity in the amygdala and this correlation was modulated by memory success for negative but not for positive or neutral stimuli. And if you look at their figure 5, it seems that this correlation was associated with forgetting for positive items. I don't think that the Ritchey findings are evidence against the NEVER model as you suggest, but are directly in line with it, and in line with this idea of valence-specific effects of arousal. This interaction between valence and arousal is referenced with respect to Ritchey et al's findings in the NEVER paper.

Small points:

Typo on page 4: “Retrieving NEUUTRAL items....

There is a citation in the text for Bowen & Kensinger 2017a and b (pg 6), but only 1 Bowen & Kensinger paper appears in the reference list.

I think you need a stronger conclusion. The paper starts off with strong aims of offering a complementary perspective, but ends rather abruptly without a summary of the main points you made in the paper.
